# Implementing strategies to improve uptake of patient-reported outcome measures (PROMs) in gender-affirming care: a mixed-methods implementation study

**DOI:** 10.1136/bmjoq-2024-002777

**Published:** 2024-04-22

**Authors:** Rakhshan Kamran, Liam Jackman, Charlie Goodwin, Anna Laws, Melissa Stepney, Conrad Harrison, Abhilash Jain, Jeremy Rodrigues

**Affiliations:** 1 Nuffield Department of Orthopaedics, Rheumatology and Musculoskeletal Sciences, University of Oxford, Oxford, UK; 2 Temerty Faculty of Medicine, University of Toronto, Toronto, Ontario, Canada; 3 Northern Region Gender Dysphoria Service, Cumbria Northumberland Tyne and Wear NHS Foundation Trust, Newcastle upon Tyne, UK; 4 Departmeny of Psychiatry, University of Oxford, Oxford, UK; 5 Department of Plastic Surgery, Stoke Mandeville Hospital, Buckinghamshire NHS Trust, UK; 6 Warwick Clinical Trials Unit, University of Warwick, Coventry, UK

**Keywords:** Patient Reported Outcome Measures, Outcome Assessment, Health Care, Implementation science, Health services research, Health Equity

## Abstract

**Importance:**

The Practical Guide to Implementing PROMs in Gender-Affirming Care (PG-PROM-GAC) is an evidence-based resource, which was developed in response to international calls for improved patient-reported outcome measure (PROM) implementation in gender-affirming care. The PG-PROM-GAC has the potential to improve PROM implementation; however, its real-world effectiveness has not yet been investigated.

**Objective:**

Investigate effectiveness and fidelity of three implementation strategies from the PG-PROM-GAC in a real-world gender clinic setting.

**Design:**

Interrupted time series mixed-methods study investigating response rates to a PROM deployed alongside implementation strategies from the PG-PROM-GAC; and open-ended feedback on the fidelity and effectiveness of implementation strategies.

**Setting:**

Participants were recruited from a National Health Service (NHS) gender clinic.

**Participants:**

Eligible participants were being seen at an NHS gender clinic for an appointment during the study period, and were invited to participate in this study via email.

**Intervention:**

Three implementation strategies from the PG-PROM-GAC deployed alongside a PROM.

**Main outcome(s) and measure(s):**

Response rates were calculated at 2-week intervals, in line with the deployment of each implementation strategy. Open-ended responses were thematically analysed by two researchers following guidance from implementation science and interpretation from Normalisation Process Theory.

**Results:**

A total of 28 participants were included in this study with a mean (SD) age of 39 (17) years. In general, participants rated education material for PROMs as the most important for PROM implementation, and accessibility options for PROMs as the second most important. Response rates to PROM completion dropped as the study progressed, as the burden of reviewing implementation strategies increased. Results were used to construct recommendations for future PROM implementation efforts.

**Conclusions and relevance:**

The PG-PROM-GAC and implementation strategy materials developed from this study (ie, educational video on PROMs co-developed with key stakeholders) can be used by clinicians, researchers and policymakers to lead PROM implementation efforts in gender-affirming care.

**WHAT IS ALREADY KNOWN ON THIS TOPIC**

- The Practical Guide to Implementing PROMs in Gender-Affirming Care (PG-PROM-GAC) is a resource which may improve patient-reported outcome measure (PROM) implementation for gender clinics. However, effectiveness of the PG-PROM-GAC in a real-world setting has not yet been investigated.

**WHAT THIS STUDY ADDS**

- This mixed-methods study conducted an interrupted time series with stepwise incremental deployment of strategies from the PG-PROM-GAC at 2-week intervals, with a total of three strategies deployed after a 2-week baseline period at a gender clinic. Response rates to a PROM and open-ended feedback on fidelity and effectiveness of implementation strategies were collected.

**HOW THIS STUDY MIGHT AFFECT RESEARCH, PRACTICE OR POLICY**

- This study presents ready-to-use implementation strategies and the PG-PROM-GAC resource to help guide PROM implementation efforts for clinical use, research and policy settings.

## Introduction

Gender-affirming care is an umbrella term used to refer to a broad range of psychosocial, hormonal and surgical care offered to transgender and gender-diverse (TGD) individuals.^1^ The primary goals of gender-affirming care may include addressing gender dysphoria^1^ and mitigating associated adverse outcomes including positively affecting mental health outcomes.^2^ It is worth noting that gender dysphoria as a term is both presumptuous and contested.^3 4^ International clinical guidelines emphasise the importance of having patient-centred gender-affirming care and using patient perspectives to guide clinical decision-making.^1 5^ Patient-reported outcome measures (PROMs) are self-report instruments that can help to improve patient-centredness of gender-affirming care with the potential to align care with patient needs.^6^

The benefits of PROMs are well researched and include improving communication between clinicians and patients,^7^ improving quality and delivery of healthcare,^8^ and supporting comparative treatment effectiveness and cost-effectiveness research.^9^ However, the potential of PROMs may not always be met due to unaddressed challenges with implementation.^10^ In some clinical settings, the rate of PROM use is as low as 1%.^11^ In order to maximise the benefits of PROMs to patients and the healthcare system, evidence-based implementation strategies are needed.^12^

The Practical Guide to Implementing PROMs in Gender-Affirming Care (PG-PROM-GAC) (online supplemental appendix 1) is a resource comprising of evidence-based implementation strategies, which might help to improve PROM uptake in gender-affirming care. The PG-PROM-GAC was developed as part of a University of Oxford doctorate led by the first author (RK), using evidence from a systematic review,^6^ qualitative study,^13^ consensus building exercise with key stakeholders, cross-sectional study and an open-ended survey. The PG-PROM-GAC was developed with input from TGD patients and gender-affirming healthcare professionals, and shown to be appropriate, acceptable and feasible in a study currently in press. However, current research on the PG-PROM-GAC has focused on its development and validation.^6 13–16^ Further research on the real-world deployment of the PG-PROM-GAC is needed to understand its effectiveness in improving PROM uptake for gender-affirming care.

10.1136/bmjoq-2024-002777.supp1 Supplementary data

The aim of this study is to deploy the PG-PROM-GAC in a real-world setting at a gender clinic and investigate its effectiveness in improving PROM uptake. The objective is to conduct an interrupted time series mixed-methods study.

## Methods

### Patient and public involvement

This study included involvement with patient and public members. Seven individuals of the TGD community were involved in confirming relevance and importance of this research, and confirming applicability of findings. Patient and public members were recruited through local and national TGD charity organisations (ie, Gender Identity Research and Education Society), and local community support groups.

### Reporting

This study followed the Standards for Reporting Implementation Studies statement for reporting.^17^

### Study design

This study involved an interrupted time series with stepwise incremental deployment of strategies from the PG-PROM-GAC at 2-week intervals, with a total of three strategies deployed after a 2-week baseline period, such that the study window lasted 8 weeks overall. Specifically, in weeks 1–2 of the study period, participants were not sent any implementation material and were invited to solely complete the PROM. In weeks 3–4, participants were sent materials related to the first implementation strategy (educational information), along with the PROM. In weeks 5–6, participants were sent educational information and PROM accessibility options, along with the PROM. In weeks 7–8, participants were sent educational information, PROM accessibility options and contact information for support with PROM completion, along with the PROM. At the end of the 8-week study period, participants were invited to complete an open-ended survey on the implementation effectiveness of strategies deployed. Different respondents were seen throughout the 8-week study period. If a participant was seen in clinic multiple times during the study period, they were invited to complete the PROM and participate in this study for their first visit.

### Implementation context, site, eligibility criteria and sample

The clinical context for the implementation strategies deployed in this study is adult (>18 years of age) gender-affirming care, though as previously discussed, these implementation strategies have wider significance for youth gender-affirming care in the UK, which is currently stagnating and mired in misinformation.^18^ Gender-affirming care is delivered through gender clinics in the UK National Health Service (NHS). However, gender-affirming care is not fully available to TGD people internationally, and many individuals face discrimination and other challenges when attempting to access gender-affirming care.^19^ The site for this study was the Northern Region Gender Dysphoria Service (NRGDS), located in Newcastle upon Tyne, UK. Personnel involved with implementation include the lead researcher (RK), assistant psychologist at the NRGDS (CG) and the lead consultant clinical psychologist at the NRGDS (AL). All individuals being seen at the NRGDS between October and December 2023 for an appointment were eligible for participation in this study. Individuals were invited by email to participate in this study by the assistant psychologist, and those who consented to participate were included.

### Implementation strategy

The implementation strategies deployed in this study were from the PG-PROM-GAC. The PG-PROM-GAC consists of two sections, one section presenting patient-relevant PROM implementation strategies, and another section presenting healthcare professional-relevant PROM implementation strategies. The top three patient-relevant strategies from the PG-PROM-GAC (education about PROMs, enhanced accessibility options for PROMs and providing contact information for PROM support) were deployed in this study.^16^ These strategies were ranked top three based on our previous study which developed the PG-PROM-GAC, where implementation strategies were ranked according to outputs from the Consolidated Framework for Implementation Research (CFIR)-Expert Recommendations for Implementing Change tool. The implementation strategies in the PG-PROM-GAC are organised by cumulative percentage of implementation experts endorsing the implementation strategies, and with refinement from key stakeholders. Implementation strategy material were co-developed with two gender-affirming healthcare professionals (AL, CG), and refined using feedback from patient and public partners (online supplemental appendix 2). The PROM administered for this study was the Gender Congruence and Life Satisfaction Scale (GCLS).^20^ The GCLS is one of the only valid PROMs developed specifically for gender-affirming care, and the only PROM with cultural validity for the UK population.^20^ Participants at the clinic were not asked to complete the GCLS before participation in this study. The top three strategies from the PG-PROM-GAC were deployed to patients being seen at the NRGDS from October to December 2023, alongside the GCLS. Participants were invited to complete the GCLS electronically via Microsoft Forms, or through a hard copy if they preferred. Justification for this was based on past research demonstrating the need for hybridised PROM implementation (both online and pen and paper options).^13^

10.1136/bmjoq-2024-002777.supp2 Supplementary data

### Outcomes and analyses

Implementation effectiveness was measured quantitatively through response rates to the GCLS on a weekly basis. Fidelity of the implementation strategies was measured through open-ended response questions sent to participants designed to cover the five key concepts of implementation fidelity.^21^ These open-ended response questions covering the five key concepts of fidelity were created using feedback from patient and public partners, and investigated: if each implementation strategy was administered as intended, participant thoughts on quality of delivery of each strategy, thoughts on if the amount of each strategy was sufficient (ie, if there was a sufficient amount of education around PROMs), thoughts on how engaging the delivery of each strategy was and thoughts on which components of each strategy are essential. At the end of the 8-week study period, all participants were sent an open-ended feedback form asking two questions on the effectiveness of PROM implementation strategies: *Was the supporting information we sent about PROMs helpful?; How do you think PROM implementation could be further improved?* Open-ended responses were thematically analysed by two researchers (RK, LJ), and interpreted in line with guidance from Normalisation Process Theory (NPT).^22^ NPT was also used to help guide and structure conclusions and recommendations from this study. NPT was selected for this study as it is a key implementation science theory which aims to understand how an innovation becomes routinised.^22^

## Results

### Characteristics of study population

In total, 250 TGD participants were eligible for participation, with a total of 223 (89%) participants consenting to take part in the study. A total of 174 participants were sent an email to complete the GCLS PROM and provide feedback on implementation strategies. Not all participants who consented to participate in the study were sent an email to complete the online form due to various reasons (eg, did not attend their appointment, email not active, appointment cancelled or were already sent the form previously). Only one participant completed the hard-copy version of a PROM, and everyone else completed the PROM electronically. Table 1 details the flow of participants for this study.

**Table 1:** Overview of flow of participants

Time period	N of patients due to be seen in clinic	N of patients agreeing to take part in the study	N of patients sent an email with PROM and feedback forms	N of patients completing the PROM and feedback forms
Weeks 1–2	50	44	38	12
Weeks 3–4	63	58	49	8
Weeks 5–6	56	49	39	5
Weeks 7–8	81	72	48	3

PROM, patient-reported outcome measure.

A total of 28 participants were included in the data collection for PROM implementation from weeks 1 to 8, with a mean (SD) age of 39 (17) years. A total of 13 (46%) participants had a male gender and 10 (36%) had a female gender. A total of 17 (61%) had a male sex assigned at birth and 11 (39%) had a female sex assigned at birth. Of the 28 participants included in this study who completed PROMs, 9 (32%) reported receiving hormonal care at the gender clinic; 5 (18%) reported receiving hormonal care and surgical care; 1 (4%) reported receiving psychosocial care; 1 (4%) reported receiving hormonal care and psychosocial care; 1 (4%) reported receiving voice therapy and hormonal care; 1 (4%) reported receiving psychosocial, hormonal and surgical care; 5 (18%) reported receiving psychosocial care, hormonal care and voice therapy; 1 (4%) reported receiving hormonal care, voice therapy and surgical care; and 1 (4%) reported receiving hormonal care, surgical care, voice therapy and psychosocial care. A total of three (11%) respondents did not respond to this question.

For the open-ended survey sent to participants who were included in the study from weeks 1 to 8, a total of 14 individuals responded. The mean (SD) age of participants for this sample was 50 (17) years. A total of eight (57%) participants had a female gender and six (43%) had a male gender. A total of 11 (79%) participants had a sex assigned at birth of male and 3 (21%) has a sex assigned at birth of female. Table 2 provides an overview of the demographic information of the sample.

**Table 2:** Demographic information of study sample

Demographic information	Frequency (%)
**TGD patient characteristics for weeks 1–8 (n=28)**	
Age, mean (SD)	39 (17)
Gender	
Female	10 (36)
Male	13 (46)
Non-binary	2 (7)
Transgender female	1 (4)
Transgender man	2 (7)
Sex assigned at birth	
Female	11 (39)
Male	17 (61)
Race	
White	28 (100)
Ethnicity	
British	24 (86)
Irish	2 (7)
Scottish	1 (4)
NR	1 (4)
**TGD patient characteristics for open-ended effectiveness survey (n=14)**	
Age, mean (SD)	50 (17)
Gender	
Female	8 (57)
Male	6 (43)
Sex assigned at birth	
Female	3 (21)
Male	11 (79)
Race	
White	14 (100)
Ethnicity	
British	14 (100)

NR, not reported; TGD, transgender and gender-diverse.

### Implementation strategies and resources

The three implementation strategies deployed for this study were the top three patient-relevant strategies from the PG-PROM-GAC (online supplemental appendix 1). These strategies included educational information about PROMs, increased accessibility options for the PROM and contact information for support with PROM completion. Online supplemental appendix 2 outlines the implementation strategy materials deployed. The assistant psychologist at the gender clinic (CG) was involved with overseeing PROM implementation, including emailing participants and responding to questions from participants.

### Response rates

The response rate to the PROM for weeks 1–2 (no implementation material support) was 32%, for weeks 3–4 (education information provided): 16%, weeks 5–6 (education information support and PROM accessibility options provided): 13%, and weeks 7–8 (educational information, PROM accessibility options and contact information for support with PROM completion provided): 6%. The response rate to the final open-ended feedback form sent to eligible participants was 50%.

### PROM scores

There were no missing data for PROM responses from the 28 participants. For each item on the GCLS PROM completed by participants, higher scores indicate positive health outcomes, with response options scored on a 5-point Likert scale. The GCLS PROM can be calculated to provide a global score from all 38 items in the scale, or subscale scores representing various constructs, or according to two clusters representing gender congruence and gender-related mental well-being and life satisfaction.

For the global scores of participants, these ranged from 2.5 to 4.8. The mean (SD) global score for all participants was 3.2 (0.5). The mean (SD) score among all participants for the cluster representing gender congruence was 3.1 (0.6), with a score range from 2.1 to 4.9. For the cluster representing gender-related mental well-being and life satisfaction, the mean (SD) score among participants was 3.3 (0.5), with ranges of scores between 2.1 and 4.7. Table 3 displays PROM scores for the sample based on each subscale.

**Table 3:** GCLS scores by subscale for study population

Subscale	Mean (SD) score	Score range
Genitalia	3.1 (1)	1–4.8
Chest	3.4 (0.7)	2.3–5
Other secondary sex characteristics	3 (1.3)	1–5
Social gender role recognition	2.9 (0.6)	1.75–4.75
Physical and emotional intimacy	3.1 (0.7)	1.75–4.75
Psychological functioning	3.5 (0.9)	1.6–4.7
Life satisfaction	3.1 (0.5)	2.6–4.9

GCLS, Gender Congruence and Life Satisfaction Scale.

### Fidelity of implementation strategies

In general, participants mentioned that the implementation strategy materials were helpful, high quality, contained a sufficient amount for each implementation strategy, were engaging and contained essential information. For the educational information provided, participants spoke about how both the video and written educational information on PROMs was helpful:

I thought this was good. I thought the video was engaging. I thought the link to the strategy document was interesting in that the references were as long as the summary. I think it’s [educational information] all important but not sure what’s essential. (Male, age 60)

The educational information was clear and succinct. (Male, age 38)

The educational information definitely should be widely available! Anything that promotes better understanding all around! (Male, age 65)

More information could be included in the online form to save switching between the form and emails. What was here was engaging though, everything that was included in the educational information seemed essential. (Female, age 38)

For the information about adaptability for PROMs, participants also provided overall positive comments for the five elements of fidelity for implementation material.

I am all for it [information about adaptability]. (Male, age 27)

Thank you for this information about adaptability. It was a pointer in the right direction and very informative and useful. I am happy to get this level of support! Just knowing that my opinions of myself matter! (Male, age 65)

The information about adaptability was good, concise, and to the point. All of it is essential. (Female, age 38)

Regarding the contact information provided to help with PROM completion, some participants spoke to the implementation resources positively:

I was happy with the contact information the quality is good, being thorough and logically presented. I thought the information was sensitively and considerately presented. (Female, age 69)

All of the contact information is necessary to gauge through support needed. (Female, age 51)

One participant offered comments on ways to improve the contact information provided. The participant spoke about how they would likely not use the contact information as it would create additional burden.

I am not going to call you part way through filling in an online form, it’s way too much effort for me. (Female, age 37)

The above quote underscores the need for clarity with forms, especially as some people may not want to engage with contact information on forms.

### Effectiveness of implementation strategies

When asked if the implementation strategies were helpful to participants and areas for improvement, most indicated that the information was helpful. In particular, the educational information and information about adaptability were generally supported by participants. Participants also appreciated the educational format of the video, which was specific to PROMs for gender-affirming care, and co-developed with the help of patient and public partners and gender-affirming healthcare professionals (available in the online supplemental appendix 2).

The educational information was helpful, especially the video. It was sufficient for my level of competence. (Male, age 60)

The information about adaptability was helpful and generally good. The educational information was good and sufficient. Education is the key to service improvement. (Female, age 57)

I think the [implementation materials] covered all valid points. (Male, age 71)

The implementation materials are a great concept and assistance to all. (Male, age 71)

However, one participant mentioned suggestions to amend the implementation strategy materials on contact information for support:

This [contact information for support with PROMs] seems helpful to me, but rather than individual contact information, perhaps it could say ‘team’ rather than individual? (Female, age 60)

A potential reason and interpretation behind the above comment is that some people may prefer to have an individual to contact, whereas other people may prefer to seek support from a ‘team’ setting.

## Discussion

This study investigates the effectiveness of three implementation strategies from the PG-PROM-GAC in improving PROM implementation at an NHS gender clinic. The results from this study can be used to help guide future deployment of the PG-PROM-GAC and refine the resource for widespread clinical use for gender-affirming care, and potentially other clinical settings.

While open-ended data from this study demonstrated the fidelity of implementation strategies deployed, as this study progressed, quantitative response rates to PROM completion fell. The lowest response rates were present when all three implementation strategies (education about PROMs, adaptability options for the PROM and contact information) were deployed. There are a few possible reasons for this. First, the lowest PROM response rates were present when participants were sent the greatest number of email attachments (three email attachments representing each implementation strategy). In a previous qualitative study, a key theme relevant for participants for gender-affirming care PROM implementation was reducing participant burden.^13^ It is possible that reviewing implementation strategy materials contributed to burden for participants and lower response rates. Beyond the burden of the number of implementation strategies to review, there was also the burden with the feedback questions to complete at the end of the PROM. Participants were asked several open-ended response questions to cover the concept of implementation fidelity, which were marked as required on Microsoft Forms. The number of open-ended response questions to complete increased with each 2-week study period, to correspond with receiving feedback for each implementation strategy deployed (three implementation strategies deployed for the final 2 weeks and increased numbers of open-ended feedback questions to cover perspectives on each of these strategies). It is possible that the burden of completing the open-ended responses following PROM completion contributed to non-response. Another possible reason is the potential impact of the holiday season on clinic attendance and response rates. As the study progressed, it continued into the Christmas/New Year holiday season. Specifically, weeks 7 and 8 of this study had the highest ‘missingness’ of people, with 48 emails out of 72 consented sent, a larger gap than previous weeks. It was confirmed when discussing with clinic staff that having staff go on annual leave and patients going on holiday breaks around this time could have contributed to lower engagement.

Our study is a useful way to evaluate multipronged implementation strategies, as it is efficient, reactive, stepwise and obtains detailed feedback from participant and users. This means that time and resource waste on ineffective strategy elements are minimised, and it can be pinpointed which elements of implementation worked and which did not, through gaining insight from end-users. The rationale for using an interrupted time series study design is the benefit it offers in observing longitudinal trends over time (before and after multiple implementation strategies are deployed to observe time-related patterns), and real-world generalisability (interrupted time series studies provide insights for how a resource may perform in a real-world setting, capturing the complexity and variability often encountered outside of controlled experimental conditions). As a next step for future research, we believe that a randomised controlled trial would be a useful future study design to further explore effectiveness of implementation strategies for PROMs in gender-affirming care.

The findings from this study can be used to create practical suggestions on how to improve implementation strategy deployment for the future. First, reducing burden of implementation strategy deployment. Specifically, rather than including multiple email attachments, embedding short links in the body of emails with implementation strategies could contribute to decreased burden on patients completing the PROM. Second, inviting participants to complete PROMs via email and directing participants to links at the end of the email that they can access, if they choose, to different implementation strategies may also improve implementation. Third, future feedback forms to collect data on PROM implementation can also be optimised to decrease burden; specifically, instead of having required open-ended response questions, keeping feedback optional could be one potential strategy. Another strategy includes having a brief follow-up interview (eg, 20 min) with a select number of participants which could help to better understand why people responded the way they did in the feedback forms. Future studies may seek to employ multiarm study designs with a control group to investigate the impact of potential confounders (ie, holidays).

This study was guided using implementation science guidance, including NPT^22^ as an overarching theory and the CFIR, which was used to develop the PG-PROM-GAC.^23^ Little to no research on gender-affirming care PROM implementation is currently available, especially with regard to the effectiveness of implementation strategies to improve PROM uptake. However, in other clinical fields, there is some overlap with the findings from this study and strategies which are also highly relevant for improving PROM uptake. The present study found that educational information, specifically video format, was highly important for improving PROM uptake, and this is supported by other studies which also demonstrate the importance of PROM education.^24 25^ PROM accessibility is also an important consideration mentioned in current literature.^26^ The present study provides an initial guide for enhancing PROM accessibility (online supplemental appendix 2); however, more research is needed in this area to investigate how accessibility of PROMs can be optimised.

This study was conducted in partnership with an assistant psychologist (CG) who helped to oversee PROM implementation, contact patients and act as a point of support for those who needed it. Having a staff member from a gender clinic involved with PROM implementation is in line with strategies from the PG-PROM-GAC. However, it is important to note that different gender clinics may have resource limitations which prevent them from having dedicated team members involved in implementation support.^27^ Staff at gender clinics may also have varied interest/drive for patient feedback and quality improvement which could impact PROM implementation efforts.

The PROM implemented for this study was the GCLS PROM.^20^ There were no missing data for the patients who completed the PROM for this study. The GCLS is a PROM which can be implemented for gender-affirming care settings as it demonstrates suitable psychometric properties and relevant content for gender-affirming care. For a UK clinical context, it is also relevant that the GCLS underwent development with input from UK TGD individuals.^20^ Other PROMs which also might be relevant to gender clinics to implement are the Utrecht Gender Dysphoria Scale^28^ and the iTransQoL,^29^ as these PROMs were developed specifically for gender-affirming care. The PG-PROM-GAC and implementation strategies were designed to supplement any PROM a clinic chooses to implement for their gender-affirming care setting and thus the results from this study have potential generalisability for other PROMs which might be implemented by a gender clinic, and potentially for paediatric gender-affirming care as well. Each of the implementation strategy information deployed in this study is present in the appendix and can be used by clinicians, researchers and policymakers for their gender-affirming care setting. These materials can also be adapted for local implementation contexts (ie, specific gender clinics) for gender-affirming care PROM implementation.

There are strengths and limitations of this study to consider. First, a key strength is this study investigated effectiveness in a real-world UK NHS gender clinic setting of PROMs and implementation strategies. This is novel for the scientific field of gender-affirming care, and the results from this study can be used to help inform service improvements for gender clinics. Second, a range of participants representing diverse ages and gender identities, and receiving diverse care (ie, psychosocial, hormonal and surgical care) were included. Third, this study investigated effectiveness through guidance from established implementation science methods, which help to ensure the methodological rigour and relevance of results for an implementation context. A key limitation of this study is the study design did not employ a control group, so the impact of confounders (holidays, etc) is unknown. A stronger design for future research would be having patients randomised to receiving different or no implementation strategies. In addition, this study included a lack of racial and ethnic diversity in the study sample. Future research should seek targeted recruitment efforts for racial and ethnic trans minorities and investigate PROM implementation for these populations. This is critically needed because TGD individuals of colour experience increased barriers to gender-affirming healthcare.^30 31^ Second, the open-ended feedback from participants was limited in richness. A potential reason for this is survey fatigue as participants had to review implementation strategy material, complete the GCLS PROM and then answer several open-ended questions on their thoughts. Future research should seek to employ one-on-one interviews or focus groups to gather feedback on implementation strategies for in-depth and richer participant perspectives. The results from this study could be used to guide development of interview guides for future qualitative studies aimed at gathering in-depth and rich participant perspectives. Finally, future research should also seek to explore PROM implementation among paediatric gender-affirming care and identification of which PROMs are most suitable to this population.^32^

## Conclusion

This study presents findings on the effectiveness of three strategies aiming to improve PROM uptake for gender-affirming care. In general, educational information that supports PROM completion, options for PROM adaptability and having contact information for support were mentioned as the most helpful for PROM implementation for gender-affirming care patients. Educational information about PROMs was viewed as the most helpful and resulted in greater PROM completion. The results and implementation strategy material provided from this study can be used by clinicians, researchers and policymakers to lead PROM implementation initiatives. The material can also be tailored for local gender clinic contexts to improve PROM implementation.

## Data Availability

Data are available upon reasonable request. Data are available upon reasonable request to the corresponding author and with a data transfer agreement, if applicable.
