# Supplementary material for: Implementing strategies to improve uptake of patient-reported outcome measures (PROMs) in gender-affirming care: a mixed-methods implementation study
Source: BMJ Open Qual. 2024 Apr 22;13(2):e002777. doi: 10.1136/bmjoq-2024-002777 (PMC11043758; doi:10.1136/bmjoq-2024-002777)
Supplement: Supplementary data [file bmjoq-2024-002777supp001.pdf]

Practical Guide to Implementing PROMs in Gender-Affirming Care

(PG-PROM-GAC)

EVIDENCE-BASED STRATEGIES TO IMPROVE PROM UPTAKE

NOV 2023 VERSION

Table of Contents

HOW TO USE THIS GUIDE 3

INTRODUCTION TO PROMS 4

PATIENT-RELEVANT STRATEGIES 5-6

HEALTHCARE PROFESSIONAL-RELEVANT STRATEGIES 7-9

REFERENCES 10

How to Use this Guide

This guide details patient- and healthcare professional-relevant strategies for implementing PROMs in gender-affirming care. The strategies are organised into two tables: one table outlines patient-relevant strategies, and the other outlines healthcare professional-relevant strategies.

The rows in both tables detail a PROM implementation strategy created using evidence from a systematic review [1], qualitative study [2], iterative refinement with patients and gender-affirming healthcare professionals [3], and feedback from a diverse sample of patients and healthcare professionals [4, 5].

You can select the implementation strategies that best apply to your setting. To maximise PROM implementation, we suggest that you tailor strategies to suit your unique setting. We also suggest that you consider using strategies together rather than viewing them as separate entities.

This guide can be printed and distributed to staff at your clinic, or to patients in your advisory group, if you have one. This guide can be used to guide a staff meeting on implementing PROMs for your gender-affirming care setting. The strategies in this guide can also be used as a checklist to ensure your clinic is maximising the potential for PROM implementation in your setting.

If you have any questions about the guide, please contact Rakhshan Kamran at rakhshan.kamran@hertford.ox.ac.uk.

Introduction to PROMs

Patient-reported outcome measures (PROMs) are questionnaires that measure how people feel and function [6]. They can be used to measure health outcomes so that healthcare professionals can better understand the patient perspective as it relates to their health. The benefits of PROMs are extensively researched and include:

- Improving communication between patients and healthcare professionals [7]
- Improving patient satisfaction [8]
- Improving health outcomes [9, 10]

For gender services, PROMs can guide shared decision-making, challenge bias where appropriate, track quality of care, and provide data to enable service improvements [1]. PROM data can also help compare between treatments and show which treatments are most effective [11]. This data can be used to show the need for additional funding for necessary services that improve patient outcomes.

Over 200 PROMs for adult gender-affirming care [1], and 38 PROMs for paediatric gender-affirming care [12] have been identified. This guide can be used to help implement a PROM most suitable for your setting. A few examples of PROMs include: the Gender Congruence and Life Satisfaction Scale (GCLS) [13], and the Utrecht Gender Dysphoria Scale (UGDS) [14].

Linked is a short video on PROMs: https://youtu.be/MjCjkoUPH2k

Patient-Relevant Strategies

Have educational material (mixture of videos, animations, written information) accessible which explains: what PROMs are, why they are being implemented, how they may benefit patient care, how they work, how data will be handled, and that care access will not be jeopardised with PROM completion. Care should be taken to ensure material is not too onerous. Coproduce educational material with service users to help with accessibility and increase engagement.

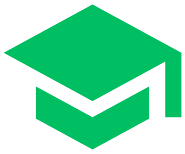

Ensure the PROM selected for implementation can adapt to patient needs (i.e., large-print, high contrast versions, different languages).

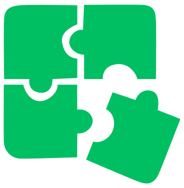

Have contact information provided of organisations or key individuals who may be able to support patients to complete PROMs (i.e., Citizens Advice, Support Worker, Assistant Psychologist).

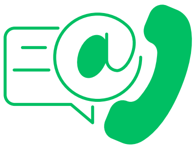

Confirm when patients would prefer to complete PROMs (i.e., before a clinic appointment, after a clinic appointment, in between appointments), and where they would prefer to complete PROMs (i.e., at home, in clinic) prior to having a PROM sent to them. Also confirm how patients would like to receive communication about completing PROMs (such as reminders) (i.e., through email, text message, post).

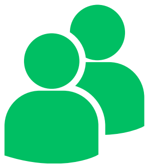

Ask patients for feedback on a regular basis (e.g., annually) for how PROM implementation is going and suggestions for improvement. Seek permission from patients prior to asking for feedback on PROM implementation. Where possible, gather input from patients at service evaluations in conjunction with PROM implementation feedback. Ensure patient feedback is from diverse populations.

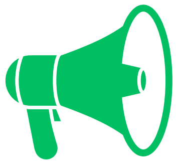

Confirm who patients would like PROM data to be shared with. Allow patients to choose levels of data usage and sharing as part of the consent process (i.e., 'I do not consent for you to use my data for research use, but you can use it for service level feedback and for my clinician to see if I am in distress').

Have a dedicated and private space to complete the PROM in clinic as an option.

Have multi-factor authentication set up for electronic PROMs so that patients can securely and remotely access their PROM and so that it cannot be accessed by unintended recipients.

Conduct an information session specifically about PROM completion and data use so patients can speak/air their views with clinicians/assistants/peer support about any questions or misgivings.

Potentially implement a parallel system for monitoring waiting list patients and outcomes resulting from waiting lists.

Have peer support staff available to contact if PROM completion is difficult. Also consider whether and how patients can access a peer support worker who is similar to the patient (i.e., age, neurodivergent, ethnicity). This may mean some of the support is provided remotely or more ad-hoc and the acceptability of this should be ascertained and led by patients. If the PROM distress falls beyond the scope of peer support services, work in collaboration with third sector organisations like LGBT switchboard or crisis mental health services.

Kamran R, *et al. BMJ Open Qual* 2024; 13:e002777. doi: 10.1136/bmjoq-2024-002777

Healthcare Professional-Relevant Strategies

Identify and prepare implementation champions who can help to oversee and be a point of support for PROM implementation in gender clinics. This may include Identifying and involving staff members (i.e., administrative staff, assistant psychologists) who can help to oversee PROM implementation.

Collect feedback on PROM implementation from healthcare professionals. Have feedback collection be part of an overall feedback system.

Develop and provide educational material to healthcare professionals on what PROMs are, why they are being implemented, how they may benefit service provision, how scoring works, and how data will be handled. Use a variety of formats such as videos, animations, written materials, and information sessions. Co-produce educational material with healthcare professionals to increase acceptability and engagement. Address staff responsibility for both healthcare improvement and integrity with data processing and collection. Aim to have material communicated in a ‘common language’ and part of a therapeutic strategy.

Capture and share local knowledge between clinics on how PROM implementation is going.

Kamran R, *et al. BMJ Open Qual* 2024; 13:e002777. doi: 10.1136/bmjoq-2024-002777

Assess/confirm patient accessibility needs to adapt PROMs as needed (i.e., large-print, high contrast versions, providing overlays, different languages).

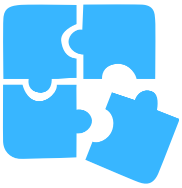

Inform higher-level leaders (i.e., senior managers) of the PROM implementation strategy for higher-level buy in.

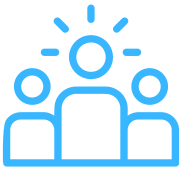

Involve local organisations as points of support to aid PROM implementation (e.g., Citizens Advice as a point of support to patients who may need help filling in a form, ethnically diverse organisations). Survey local organisations to see if they would be willing to be involved and if they have the knowledge required to support a gender-affirming care PROM implementation effort.

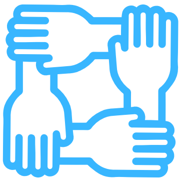

Involve local patient advisory groups as points of contact to provide support on PROM implementation. This could include tailoring PROM implementation strategies to your clinic in partnership with service users.

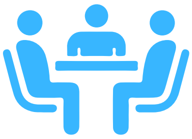

Organise staff meetings aimed at identifying a PROM to implement which is not burdensome (i.e., not too lengthy or complex to score, has a computerised adaptive test option) and formalising the PROM implementation plan. Also, organise a meeting with service users to identify measures which would be acceptable to them.

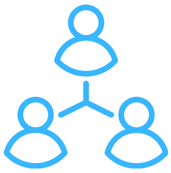

Develop a formal implementation blueprint for the clinic on PROM implementation.

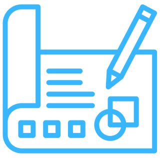

Provide ongoing engagement with patients to facilitate dialogue about how PROM responses are used to improve care.

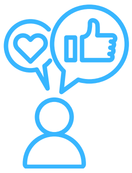

Develop academic partnerships to help facilitate PROM implementation and interpretation when using PROMs for research.

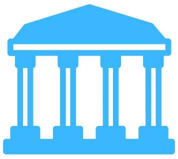

Have PROM responses linked to the electronic medical record so they are accessible online. Ensure patient control over data access and where patients consent.

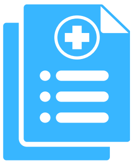

Develop a process to handle critical PROM responses and feedback. Have details of this process available for patients.

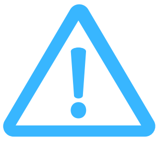

Kamran R, *et al. BMJ Open Qual* 2024; 13:e002777. doi: 10.1136/bmjoq-2024-002777

References

1. Kamran R, Jackman L, Chan C, et al. Implementation of Patient-Reported Outcome Measures for Gender-Affirming Care Worldwide: A Systematic Review. JAMA Netw Open. 2023;6(4):e236425. Published 2023 Apr 3. doi:10.1001/jamanetworkopen.2023.6425
2. Kamran R, Jackman L, Laws A, et al. Patient and Healthcare Professional Perspectives on Implementing Patient-Reported Outcome Measures in Gender-Affirming Care: A Qualitative Study. BMJ Open Quality. In press.
3. Kamran R, Jackman L, Laws A. A Practical Guide to Implementing PROMs in Gender-Affirming Care: An Implementation Study. Under Review.
4. Kamran R, Jackman L, Laws A. A Practical Guide to Implementing Patient-Reported Outcome Measures in Gender-Affirming Care: Evaluating Acceptability, Appropriateness, and Feasibility. Under Review.
5. Kamran R, Jackman L, Laws A. Patient and Healthcare Professional Perspectives on the Practical Guide to Implementing PROMs in Gender-Affirming Care (PG-PROM-GAC): Analysis of Open-Ended Responses from Patients and Healthcare Professionals. In Submission.
6. Weldring T, Smith SM. Patient-Reported Outcomes (PROs) and Patient-Reported Outcome Measures (PROMs). Health Serv Insights. 2013;6:61-68. Published 2013 Aug 4. doi:10.4137/HSI.S11093
7. Snyder CF, Blackford AL, Aaronson NK, et al. Can patient-reported outcome measures identify cancer patients' most bothersome issues?. J Clin Oncol. 2011;29(9):1216-1220. doi:10.1200/JCO.2010.33.2080
8. Chen J, Ou L, Hollis SJ. A systematic review of the impact of routine collection of patient reported outcome measures on patients, providers and health organisations in an oncologic setting. BMC Health Serv Res. 2013;13:211. Published 2013 Jun 11. doi:10.1186/1472-6963-13-211
9. Nelson EC, Eftimovska E, Lind C, Hager A, Wasson JH, Lindblad S. Patient reported outcome measures in practice. BMJ. 2015;350:g7818. Published 2015 Feb 10. doi:10.1136/bmj.g7818
10. Basch E, Deal AM, Dueck AC, et al. Overall Survival Results of a Trial Assessing Patient-Reported Outcomes for Symptom Monitoring During Routine Cancer Treatment. JAMA. 2017;318(2):197-198. doi:10.1001/jama.2017.7156
11. Black N. Patient reported outcome measures could help transform healthcare. BMJ. 2013;346:f167. Published 2013 Jan 28. doi:10.1136/bmj.f167
12. Jackman L, Chan C, Jacklin C, et al. Patient-Reported Outcome Measures for Pediatric Gender-Affirming Care: A Systematic Review. Under Review.
13. Jones BA, Bouman WP, Haycraft E, Arcelus J. The Gender Congruence and Life Satisfaction Scale (GCLS): Development and validation of a scale to measure outcomes from transgender health services. Int J Transgend. 2018;20(1):63-80. Published 2018 Apr 26. doi:10.1080/15532739.2018.1453425
14. McGuire JK, Berg D, Catalpa JM, et al. Utrecht Gender Dysphoria Scale - Gender Spectrum (UGDS-GS): Construct validity among transgender, nonbinary, and LGBQ samples. Int J Transgend Health. 2020;21(2):194-208. Published 2020 Apr 11. doi:10.1080/26895269.2020.1723460
