# Supplementary material for: Implementing strategies to improve uptake of patient-reported outcome measures (PROMs) in gender-affirming care: a mixed-methods implementation study
Source: BMJ Open Qual. 2024 Apr 22;13(2):e002777. doi: 10.1136/bmjoq-2024-002777 (PMC11043758; doi:10.1136/bmjoq-2024-002777)
Supplement: Supplementary data [file bmjoq-2024-002777supp002.pdf]

Educational Material on PROMs

Patient-reported outcome measures (PROMs) are questionnaires that measure how people feel and function[1]. They can be used to measure health outcomes so that healthcare professionals can see how you are doing. The benefits of PROMs are extensively researched and include:

- Improving communication between patients and healthcare providers[2]
- Improving patient satisfaction[3]
- Improving health outcomes[4,5]

For gender services, PROMs have the ability to provide evidence and measurement of quality and care received, guide shared decision-making, challenge bias where appropriate, and provide data to enable service improvements[6]. PROM data can also help us compare between treatments and show us which treatments are most effective[7]. This data can be used to show the need for additional funding for necessary services that improve patient outcomes.

Data from PROMs are securely and carefully handled. All data will be anonymised, and secured on an encrypted server.

Linked is a short video on PROMs: https://youtu.be/MjCjkoUPH2k

References:

1. Weldring T, Smith SMS. Patient-reported outcomes (PROs) and patient-reported outcome measures (PROMs). *Health Serv Insights*. 2013;6:61-68.
2. Snyder CF, Blackford AL, Aaronson NK, et al. Can patient-reported outcome measures identify cancer patients' most bothersome issues? *J Clin Oncol*. 2011;29(9):1216-1220. doi:10.1200/JCO.2010.33.2080
3. Chen J, Ou L, Hollis SJ. A systematic review of the impact of routine collection of patient reported outcome measures on patients, providers and health organisations in an oncologic setting. *BMC Health Serv Res*. 2013;13:211. doi:10.1186/1472-6963-13-211
4. Nelson EC, Eftimovska E, Lind C, Hager A, Wasson JH, Lindblad S. Patient reported outcome measures in practice. *BMJ*. 2015;350:g7818. doi:10.1136/bmj.g7818
5. Basch E, Deal AM, Dueck AC, et al. Overall survival results of a trial assessing patient-reported outcomes for symptom monitoring during routine cancer treatment. *JAMA*. 2017;318(2):197-198. doi:10.1001/jama.2017.7156
6. Kamran R, Jackman L, Chan C, et al. Implementation of Patient-Reported Outcome Measures for Gender-Affirming Care Worldwide: A Systematic Review. *JAMA Netw Open*. 2023;6(4):e236425. Published 2023 Apr 3. doi:10.1001/jamanetworkopen.2023.6425
7. Black N. Patient reported outcome measures could help transform healthcare. *BMJ*. 2013;346:f167. doi:10.1136/bmj.f167

Accessibility Options for PROM

The GCLS PROM is provided in the document below.

The GCLS PROM below can be edited to change the font size and colour based on your preference.

If you would like to use the “Immersive Reader” feature, please follow the below steps:

1. In your document, select **View** > **Immersive Reader**.
2. On the **Immersive Reader** tab, select **Read Aloud**.
3. To exit **Immersive Reader**, select **Close Immersive Reader**.

If you would like to have the questions from the PROM read aloud to you, you can use the following resource and copy-and-paste the questions from the PROM to have it read aloud to you:

https://www.naturalreaders.com/online/

You can email the completed form back to us once you are finished. Thank you for helping to improve our service.

Contact Information for PROM Support

If you would like additional support with completing the PROM, you can contact the individuals below who may be able to help:

Contact for Peer Support: NRGDSPeerSupport@cntw.nhs.uk

Contact for Citizens Advice: https://www.citizensadvice.org.uk/about-us/contact-us/contact-us/contact-us/
